# Optical imaging of the small intestine immune compartment across scales

**DOI:** 10.1038/s42003-023-04642-3

**Published:** 2023-03-31

**Authors:** Arielle Louise Planchette, Cédric Schmidt, Olivier Burri, Mercedes Gomez de Agüero, Aleksandra Radenovic, Alessio Mylonas, Jérôme Extermann

**Affiliations:** 1 Institute of Bioengineering, École Polytechnique Fédérale de Lausanne (EPFL), 1015 Lausanne, Switzerland; 2 HEPIA/HES-SO, University of Applied Sciences of Western Switzerland, Rue de la Prairie 4, 1202 Geneva, Switzerland; 3 BioImaging & Optics Platform, Ecole Polytechnique Fédérale de Lausanne (EPFL), 1015 Lausanne, Switzerland; 4 Host-microbial interactions group, Institute of Systems Immunology, Max Planck research group, University of Würzburg, Würzburg, Germany; 5 Mucosal Immunology Group, Department for Biomedical Research, University of Bern, Bern, Switzerland

**Keywords:** Ileum, Optical imaging

## Abstract

The limitations of 2D microscopy constrain our ability to observe and understand tissue-wide networks that are, by nature, 3-dimensional. Optical projection tomography (OPT) enables the acquisition of large volumes (ranging from micrometres to centimetres) in various tissues. We present a multi-modal workflow for the characterization of both structural and quantitative parameters of the mouse small intestine. As proof of principle, we evidence its applicability for imaging the mouse intestinal immune compartment and surrounding mucosal structures. We quantify the volumetric size and spatial distribution of Isolated Lymphoid Follicles (ILFs) and quantify the density of villi throughout centimetre-long segments of intestine. Furthermore, we exhibit the age and microbiota dependence for ILF development, and leverage a technique that we call reverse-OPT for identifying and homing in on regions of interest. Several quantification capabilities are displayed, including villous density in the autofluorescent channel and the size and spatial distribution of the signal of interest at millimetre-scale volumes. The concatenation of 3D imaging with reverse-OPT and high-resolution 2D imaging allows accurate localisation of ROIs and adds value to interpretations made in 3D. Importantly, OPT may be used to identify sparsely-distributed regions of interest in large volumes whilst retaining compatibility with high-resolution microscopy modalities, including confocal microscopy. We believe this pipeline to be approachable for a wide-range of specialties, and to provide a new method for characterisation of the mouse intestinal immune compartment.

## Abstract (web summary)

A workflow for 3D characterization of the mouse small intestine with optical projection tomography allows the identification of sparsely-distributed regions of interest in large volumes while retaining compatibility with high-resolution microscopy modalities.

## Introduction

The intestine forms an interface between the external environment and the rest of the body, fulfilling many essential functions in the process. Among these are immune system education, and the regulation of the microbiome—which are incidentally interdependent^1,2^. Indeed, we now know that the microbiome is necessary for the correct education of the immune system^3,4^ and that this has long-term repercussions on intestinal immunity. As such, the gut microbiome has been linked to distinctly immune-related disorders ranging from obesity^5^ and diabetes^6^, to auto-immune^7^ and allergic diseases^8–10^. Importantly, significant advances in the aetiology of such disorders were made through studying gut structure^11–13^. For example, deficits in gut morphology and barrier permeability have been implicated in both obesity^14,15^ and diabetes^16^.

Secondary and tertiary lymphoid tissues (LT) are strategically positioned along the gastrointestinal tract to orchestrate immuno-surveillance against pathogens and invading microorganisms. These are known to develop during early life^17^, and to require stimuli from the microbiome^18,19^. Peyer’s patches (PP, secondary LT) and isolated lymphoid follicles (ILFs, tertiary LT) form key networks where microbial antigens are presented to T- and B-cells, effectively dictating both regulation and tolerance to commensals. While PPs are formed prenatally, ILFs are known to form postnatally^1^ and due to commensal bacterial cues. PPs are visible to the naked eye whereas ILFs can only be seen under magnification. Hence, new methods are continuously being developed for immuno-phenotyping and isolating immune cells from ILFs^20,21^, yet their function—deriving from their location at the interface with the microbiota— necessitates further research in their spatial context. Concurrently, the awareness of the importance of gut spatial structures demanded techniques that deliver in large-area, volumetric mapping.

Optical projection tomography (OPT) is a 3D imaging modality that is ideal for mesoscale imaging, offering broad applicability while maintaining compatibility with other microscopy techniques. With fields of view spanning from a few millimetres to 60 mm in length^22^ and full 3D volume acquisition times ranging from minutes to an hour^23^, OPT is a time- and cost-effective technique with which large-scale structural and functional parameters can be imaged. OPT has been leveraged for concatenated, functional multi-channel plant imaging^22^, functional cell proliferation imaging in zebrafish^24^, multi-orientation digital sectioning of whole mouse embryos^25^ and mouse organ imaging of the liver, pancreas^23^ and brain^26^.

Recently, we developed a multi-spectral OPT modality to image the mouse gut^27^, with a 3D resolution of 28 μm allowing the distinction of mucosal layers and villi in samples several centimetres in length. To demonstrate the wide range of applications for this method, we present a workflow that enables mesoscale observation of signal distribution throughout millimetre-long gut sections with autofluorescent contextualization, as well as the identification of regions of interest that can be characterized at higher-resolution following reverse-OPT (RevOPT) processing. We apply this workflow to visualise the immune compartment of the terminal ileum, rich in ILFs, and present the first observation of their distribution in a single acquisition spanning several millimetres of tissue. Furthermore, we confirm the lack of large organised lymphoid structures in age-matched adult 30-week old germ-free as well as adolescent 2-week old conventionally-raised mice, reflecting the microbiome and age dependence of the gut immune system development. Finally, by implementing RevOPT and subsequent confocal microscopy, we show the feasibility of tracking regions of interest (ROIs) initially selected in 3D for subsequent higher resolution imaging using traditional histology methods. This method is a powerful approach for characterizing tissues at multiple scales while providing high resolution data with ready-to-use processing pipelines optimized for the mouse gut.

## Results

### Sample preparation pipeline and label-free 3D tissue imaging

**Fig. 1:**
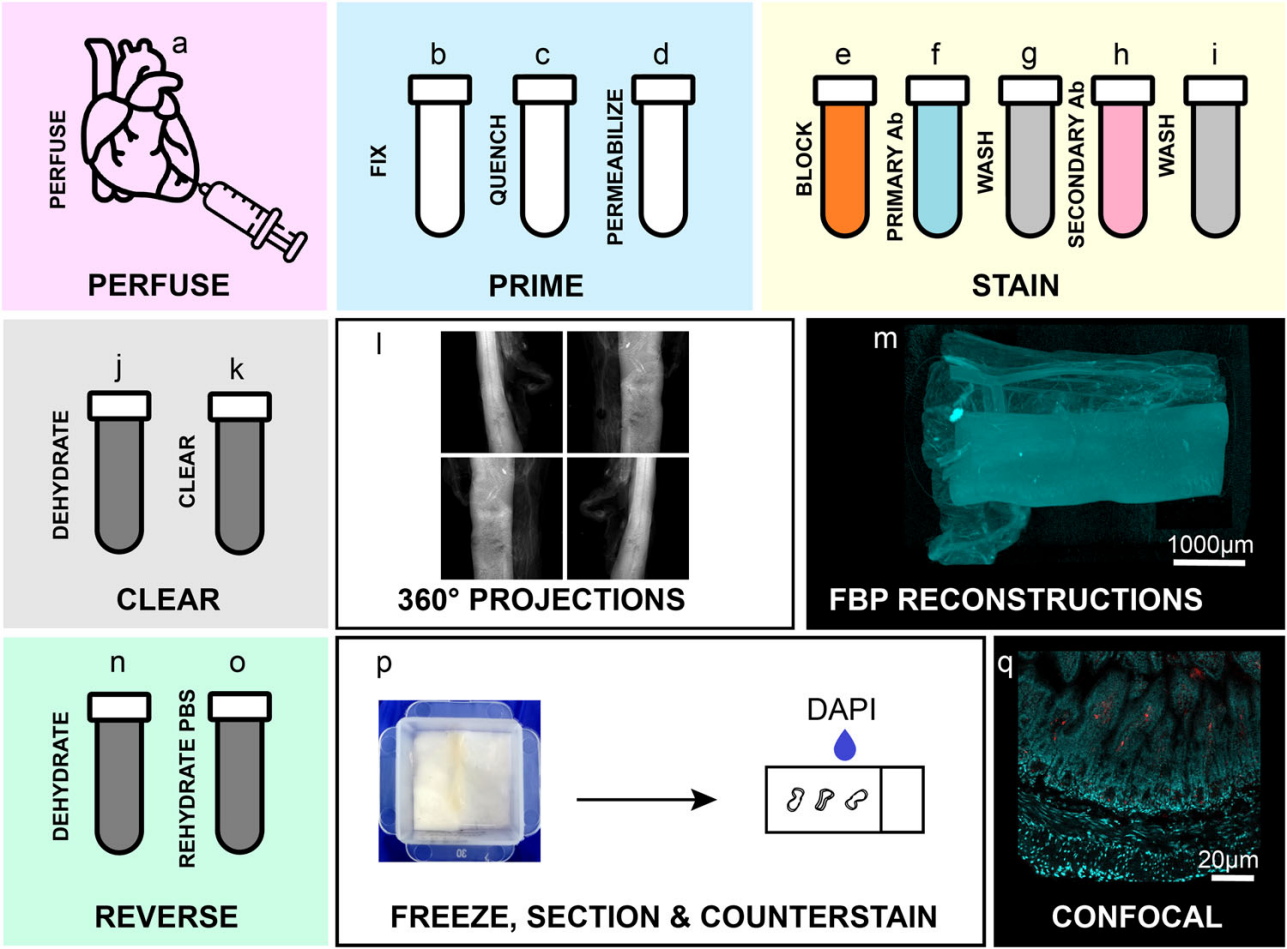
gutOPT workflow. Sample preparation and handling in four steps: sample preparation (**a**–**k**), imaging and image processing (**l**, **m**), reverse-OPT (**n**–**p**) and secondary imaging (**q**). The experimental steps for sample preparation are: transcardiac perfusion of PBS and 4% PFA (**a**); 4-hour PFA fixation (**b**); overnight autofluorescence quenching with methanol, DMSO and hydrogen peroxide 1:3:2 mixture (**c**); permeabilisation by three freeze-thaw cycles between −80 °C (1 h) and room temperature (30 min) (**d**); 24-hour tissue blocking (**e**); 48-hour primary antibody incubation (**f**); 24-hour wash (**g**); 48-hour secondary antibody incubation (**h**); 24-hour wash (**j**); 36-hour dehydration in pure methanol with two solution changes (**j**) and clearing in 1:2 ratio of benzyl alcohol and benzyl benzoate (**k**). Optical projections are acquired over a 360° rotation of the sample (**i**) and a 3D image is reconstructed using filtered back projection (**m**). OPT processing may be reversed in order to image sections of the same sample using alternative imaging modalities. To do so, samples are dehydrated in pure methanol for 36 hours (**n**), rehydrated in PBS (**o**), mounted in cryomatrix, cryo-sectioned and counterstained with DAPI (**p**). High-resolution imaging modalities such as confocal microscopy (**q**) may be applied to samples whose large-scale volume has been observed.

To achieve the multi-scale observation of spatially-distributed biological signals of interest, we developed a sample preparation pipeline that includes two imaging modalities: high-volume optical projection tomography and high-resolution confocal microscopy after RevOPT (Fig. 1). The pipeline is divided into four phases, sample preparation (Fig. 1a–k), imaging and image processing (Fig. 1l, m), RevOPT (Fig. 1n–p) and secondary imaging (Fig. 1q), spanning a duration of approximately three weeks. First, tissue preservation, autofluorescence quenching and tissue permeabilisation are performed to prepare the samples for staining. This is followed by fluorescent antibody staining of select markers. A clearing step precedes the acquisition of optical projections over a 360° sample rotation. Specifically for the intestine, we set up a system able to image a sample up to 10 cm in length and up to 5 mm in diameter. We optimised an illumination power density of 0.1 W/cm^2^ and 1200 projections spanning 360°. With a camera integration time of 0.5 seconds, this results in a total acquisition time of ~10 minutes per channel. We performed multispectral acquisitions with 415 nm and 625 nm wavelengths, spectrally filtered between 400 nm and 440 nm, and 595 nm and 645 nm respectively. Emissions were measured between 455 nm and 520 nm, and 620 nm and 650 nm respectively. Next, we used a filtered back-projection algorithm to reconstruct the projections into a 3D image as described previously^27^, taking less than 10 minutes on a dedicated terminal for full reconstruction and channel overlap. Finally, we use RevOPT to revert the sample to a state compatible with freezing in Optimal Cutting Temperature (OCT) compound, allowing for cryostat sectioning and counterstaining (Fig. 1p). In amongst several methods requiring thin sectioning including electron microscopy or single-molecule FISH, we selected confocal microscopy to image regions of interest identified by OPT with improved resolution (Fig. 1q).

**Fig. 2:**
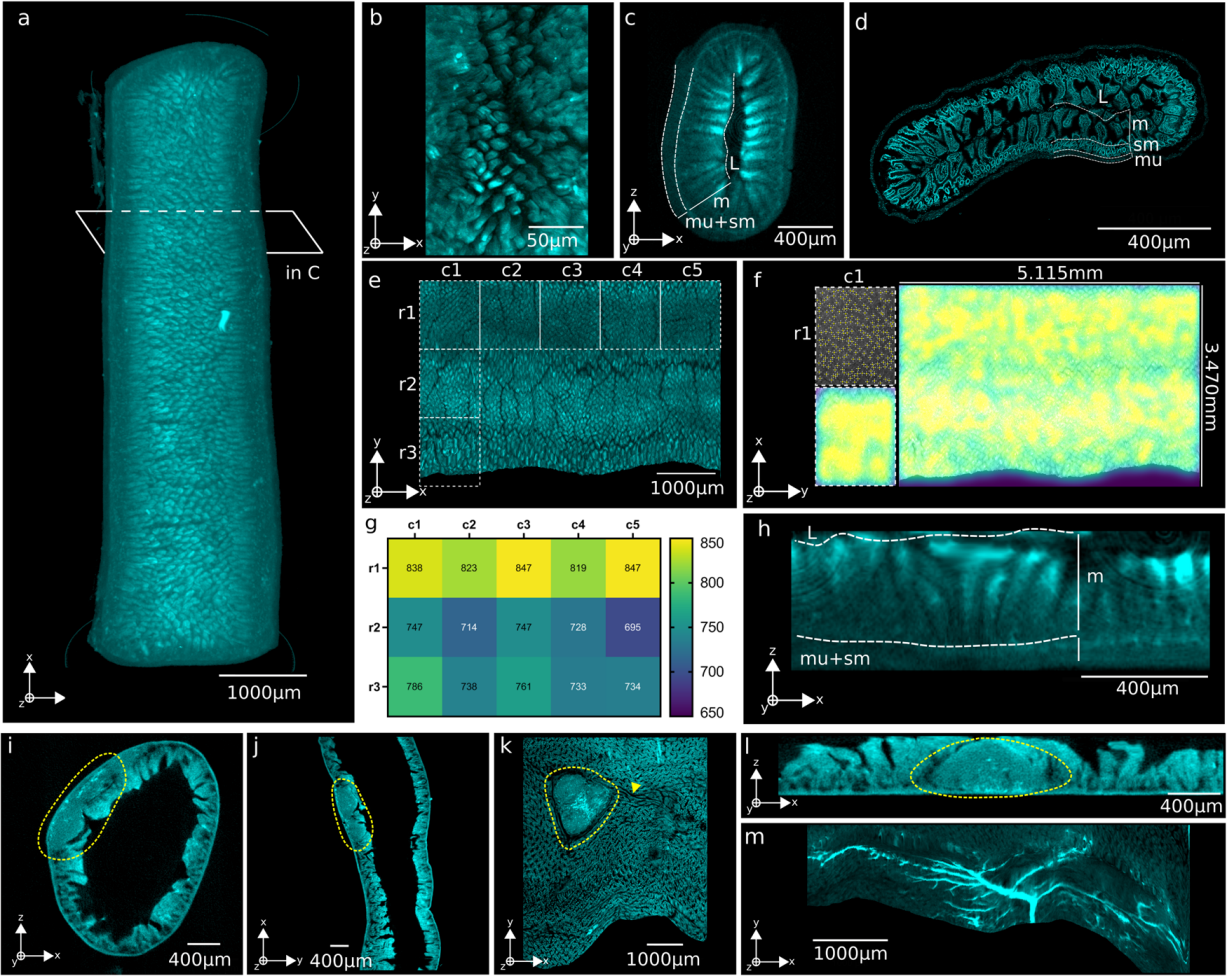
Label-free visualisation and characterization of the intestine. **a** Filtered Back Projection reconstruction from a single acquisition of gut in the autofluorescence channel. **b** Close-up of the villi structures from within the lumen. **c** Cross-sectional view of the 3D reconstruction, with visible layers labelled mu muscularis, sm submucosa, m mucosa and L lumen. **d** High-resolution tiled acquisition of the same gut sample using confocal microscopy, with concurrent layers labelled. **e** Innermost layer of the same reconstruction that was virtually unfolded^28^. **f** Unfolded image and whole gut segment quantification of villous density by Laplacian filter and the function *find maxima*. **g** Villous density quantified within sub-regions of the unfolded scan. **h** Cross-sectional view of unfolded image, with mucosal layers labelled as in **c**. **i**–**m** Images of tertiary lymphoid structures encircled in yellow from a cross-section view (**i**, **j**) or top to bottom (**l**). **k** Unfolded image reveals tri-lobular structure and possible vascularisation (arrow). **l** Straightened cross section of structure in **i**. **m** Widespread network of large vessels in an unfolded image.

Tissue autofluorescence is an inherent signal produced by extracellular matrix components and certain pigmented cell types. In OPT, autofluorescence quenching is required to reduce noise and retain targeted fluorescent signals^27^ (Fig. 1, steps **a** and **c**). However, low levels of autofluorescence enable the discrimination of the outer and inner layers of the gut when samples are illuminated at 415 nm spectrally filtered between 400–440 nm, whilst emission is collected within the range of 455–520 nm^27^. A longitudinal portrayal of the gut (Fig. 2a) provides an overview of the structures present in the tissue. In reconstructions made up of 1200 projections, well-resolved villi can be observed in 3D (Fig. 2b). When taking a cross-sectional view, the mucosal layers can be distinguished from the villi in the OPT scan (Fig. 2c; mu = muscularis, sm = submucosa, m = mucosa and L = lumen) whilst a greater resolution is achieved by confocal microscopy on the same sample having undergone RevOPT (Fig. 2d). During RevOPT, counterstaining is possible and demonstrated here by the staining of DNA with DAPI (Fig. 2d). We observe a preservation of the cross-sectional structure in OPT when comparing the virtual section (Fig. 2c) and its histological counterpart (Fig. 2d).

We implemented a virtual unfolding technique^28^ (supplementary SI Fig. 1) to observe the gut tissue from within the lumen, with sections spanning from the mucosa to the serosa (Fig. 2e, section closest to lumen). We calculated villous density by segmenting the unfolded image and finding local maxima (Fig. 2f). This can be performed for the whole tissue region or applied to smaller regions of interest to probe different areas of the tissue. We then transformed this into a quantitative visualisation of different sectors (Fig. 2g). In this healthy tissue, overall villous density is mostly homogeneous. Virtual unfolding also yields a straightened image (Fig. 2h) of the tissue cross section seen in Fig. 2c.

Virtual unfolding of 3D-reconstructed data can lead to detailed visualizations of structures that are difficult to visualise in a 3D image such as Fig. 2a or in a virtual cross-section as in Fig. 2c. We found a suspected lymphoid follicle in the autofluorescence channel (top view Fig. 2i, side view Fig. 2j), whose structural context is made clear by virtual unfolding (Fig. 2k and straightened Fig. 2l). The follicle is made up of three lobes, with a concentration of fluorescent vessels in the centre. In the areas surrounding the follicle, gaps in the villi suggests the potential presence of lymphatic vasculature. Typically, large vascular networks are difficult to observe by visualization of cross- sections. In Fig. 2m, an example of such a network is shown, highlighting the added value that processing the autofluorescence channel can bring to gut structure characterization.

### Cell-type specific signal distribution throughout the gut volume

**Fig. 3:**
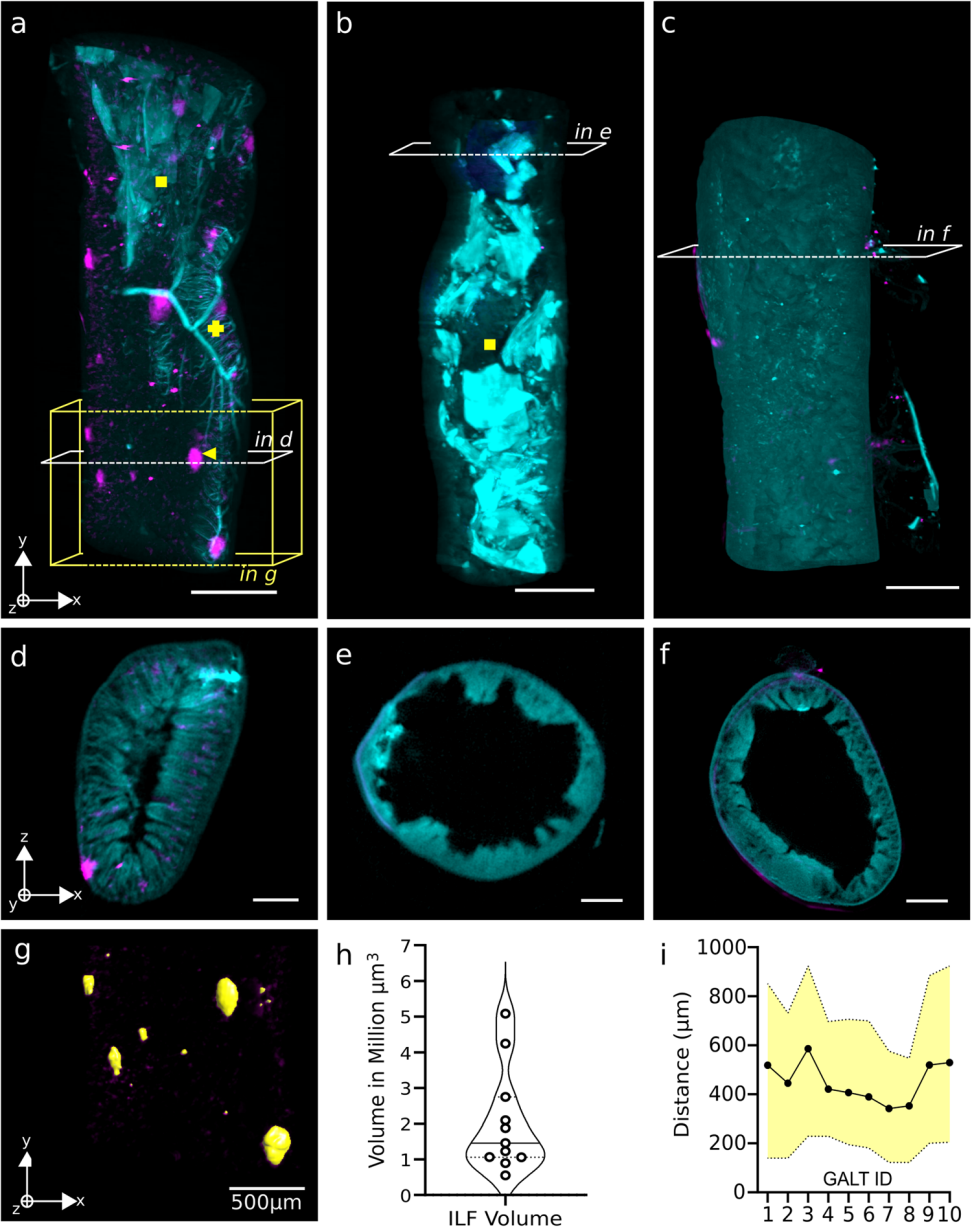
Multi-channel OPT reconstructions and segmentation of gut immune compartment. **a**–**c** Longitudinal views of two-channel OPT renders with tissue autofluorescence in cyan and CD45-positive cell clusters in magenta. Age- and microbiota-dependent development of gut-associated lymphoid tissues (GALTs) is evident in a healthy adult SPF gut (**a**) versus adolescent SPF (**b**) and adult germ-free (**c**) gut samples. Structures of interest include the positive cell clusters (arrow), vascular network (cross) and luminal dietary fibers (square). Scale bars represent 1000 μm. **d**–**f** Cross-sectional view of 3D reconstructions for the samples shown (**a**–**c**). Scale bars represent 200 μm. **g** 3D segmentation of signal from isolated lymphoid follicles (ILFs) observed in box shown in **a**. **h** Violin plot of measured volume of individual CD45-positive regions in an adult C57BL/6J mouse, measured in million-μm^3^. **i** Spatial distribution of ILFs throughout the tissue seen in **a** as measured by minimum, mean and maximum distance in μm between each follicle.

OPT can also be used for visualisation of cell types according to staining of selective markers. To demonstrate this, we chose to stain the gut immune compartment, due to its structured organisation under healthy conditions and its common deregulation in gastrointestinal diseases (e.g. Inflammatory Bowel Disease)^29^ and other systemic disorders (e.g. metabolic diseases^30,31^, autoimmunity^32^, and neurodegeneration)^33,34^. For this we stained CD45-positive cells using fluorescently-labelled antibodies. In healthy adult mice, we find immune cells interspersed at regular intervals or compartmentalised in gut-associated lymphoid tissues (GALTs) known as isolated lymphoid follicles (ILFs, Fig. 3a yellow arrow). Overlaying the autofluorescence channel reveals other adjacent structures such as blood vessels and luminal dietary fibers (Fig. 3a, cross and square respectively). In order to better visualise the three-dimensional characteristics, we assembled a movie from the reconstructed images (SI Movie 1).

It is known that age- and microbiota-dependent education of the immune system is responsible for the formation of lymphoid structures such as Peyer’s patches and ILFs^35,36^. We confirm that with OPT, we are able to identify differences in the immune cell compartments in the contexts of adolescent (14 days) SPF mice and adult (30+ weeks) germ-free mice (Fig. 3b, c respectively), compared to the old (30+ weeks) SPF mouse shown in Fig. 3a. At a young age under normal rearing conditions, no dense regions of immune cells are observed (Fig. 3b). Intestines of older, germ-free mice also exhibit reduced CD45-positive cell clusters in the mucosal layers. A two-channel cross-sectional view of these samples (Fig. 3d–f) frames the immune signal within the structured layers of the gut. An isolated lymphoid follicle is located within the sub-mucosal layer and is surrounded by smaller, less dense CD45-positive clusters in Fig. 3d. Conversely, there is no specific fluorescence in both old germ-free and adolescent SPF animals, thus indicating a lack of well-defined GALT structures in these mouse models (Fig. 3b, c, e and f).

OPT reconstructions can thus be used for broader, organ-scale characterisation of the mouse intestine. Furthermore, current 3D image processing tools allow for accurate quantification of different parameters. We segmented the ILFs in the 625 nm channel alone (Fig. 3g) and found their size ranges from approximately 1 to 5 million μm^3^ (Fig. 3h). Their spatial distribution along the small intestine is uniform, averaging at 500μm in between ILFs (Fig. 3i).

### gutOPT pipeline for multi-modal imaging and high resolution characterisation of the gut

Because sample preparation for optical projection tomography is compatible with downstream processing for additional imaging modalities, we wondered whether we could incorporate a single pipeline for imaging at different scales. To do this, we performed RevOPT (Fig. 1n, o) on the samples shown in Fig. 3 and imaged them using confocal microscopy.

**Fig. 4:**
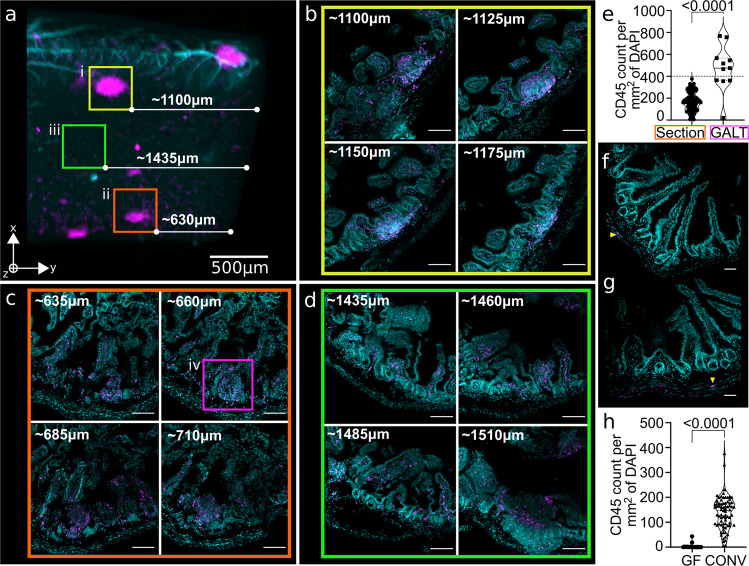
Reverse OPT for tracking of ROIs in high-resolution imaging modalities. **a** Close-up and selection of two GALT ROIs in OPT reconstruction, with calculation of distance from the end of the tissue. Regions i–iii are regions shown in **b**–**d**, with sectors i and ii containing CD45 signal and iii being empty. **b**–**d** Scale bars represent 200 μm. **b** Two-channel confocal images of reverse-OPT sections within the depth of (i) in **a**, with distance from the end of the tissue calculated based on the number of 25-μm-thick sections collected. Nuclei were stained with DAPI and CD45 signal comes from pre-OPT staining. High CD45-positive cell density regions are the ILFs visible by OPT and are surrounded by sparse CD45-positive cells. **c** Confocal depths in the second ROI (ii) selected in **a**. **d** Confocal depth in a region with no observed ILFs (iii) in **a** showing only sparse CD45 signal. **a**–**d** Images come from a healthy SPF adult mouse gut. **e** Violin plot of CD45-positive cell density within GALT region and surrounding tissue measured in sections from four gut samples. The signal density threshold above which structures are visible in OPT reconstructions is estimated at 400 fluorescent cells/mm^2^ of DAPI signal. Sections and GALTs *n* = 100 and *n* = 10 respectively. Confocal acquisitions from adult germ-free (**f**) and adolescent SPF (**g**) mice as in Fig. 3b and c respectively, with no ILF signal observed in OPT and concurrently a visibly reduced presence of CD45-positive cells. Scale bars represent 200 μm. **h** Violin plot of whole-section area density of CD45-positive cells in SPF (*n* = 75) and germ-free (*n* = 15) gut samples (example images **b**–**d** and **f**), showing significant reduction in immune cell presence in the gut lining of germ-free animals.

We used OPT to pre-select regions of interest, retraced their exact location and imaged them using a higher-resolution technique. To do this, we selected isolated lymphoid follicles in the OPT reconstruction and calculated their distance from the edge of the sample (Fig. 4a—i and ii). We then performed RevOPT and mounted the tissues in optimal cutting temperature (OCT) compound. The depth of each cryosection was used to track the localization of the ROIs. We find that the fluorescence signal was maintained from the OPT staining, and sections do not require further immuno-staining for confocal imaging. Furthermore, we find that preselected ILF regions observed by OPT are high-density cell clusters rich in CD45-positive cells (Fig. 4b, c). In both ROIs containing ILFs, the calculated distances were accurate, and the immune cell-dense regions were situated within the submucosa layer as expected from the OPT reconstructions and their known localisation^37,38^. Areas lacking GALTs in 3D (Fig. 4a—iii) only contain sparse CD45-positive cells in the lamina propria at higher resolution (Fig. 4d). By measuring the immune cell density in the whole-sectioned GALT regions, we find that the signal density threshold for visibility in OPT is approximately 400 fluorescent cells per mm^2^ of DAPI signal (Fig. 4e). Lastly, we imaged the adult germ-free samples that display no GALTs by OPT (Fig. 4f). Here, we find no CD45-positive cells along the length of the villi nor in the submucosa, confirming that no ILFs are present, and further confirming that the lack of a microbiome indeed alters the immune compartment in the gut (Fig. 4f, triangle). The number of immune cells is also significantly reduced compared to that observed in the gut of SPF mice (Fig. 4g). Thus, OPT can be used to identify specific structures and markers of interest using tissue-wide staining, and given a sufficiently dense fluorescent signal ROIs can be traced by confocal microscopy using RevOPT and cryosectioning.

## Discussion

The choice of microscopy technique for gut characterization relies on certain features of the signal of interest, including the scale, the required resolution, the need for staining, the sample preparation and its application in-vivo or ex-vivo. In studying the gut, techniques used should allow for studying villous vascularization^39^, structural integrity^28,40^, local inflammatory status^41^, and microbial community dynamics^42^. The heterogeneity of gut tissue structure and the dynamic recruitment and trafficking of cells involved in gut health makes 3D microscopy particularly adapted for this. In addition, volumetric imaging modalities are continually being developed in parallel with advanced image processing techniques^43–45^, as the weight of data grows rapidly with 3D imaging.

We describe a multi-scale and multi-modal pipeline for visualising the gut architecture and associated isolated lymphoid follicles (ILFs) at the scale of organs. We provide quantifications of volumetric sizes and spatial distribution of ILFs in adult mice throughout centimetre lengths of mouse intestine. We leverage the 3-dimensional nature of OPT data to facilitate the observation of vascular networks in the submucosa, as shown by virtual unfolding. As proof-of-concept, we have evidenced the requirement of the microbiome for the maturation of ILFs, and during development. Finally, we have incorporated a technique—which we have called RevOPT—for higher-resolution imaging of ROIs selected in 3D. This technique bridges imaging of tissues at the organ and histology level, and allows quantitation at different scales. While this methodology requires specialized manipulation lasting up to three weeks, the gain in information and wide field of view is attractive for studying the distribution and localisation of distinct cellular structures.

As a technique, OPT has certain disadvantages when compared to other imaging modalities. Micro-computed tomography (micro-CT) can be used to produce high resolution 3D images of naturally opaque structures such as that of the intestine^46^, yet is largely less laborious than OPT because no clearing of tissues is required. It uses X-ray detection scintillators and rotational scanning to produce images, and similarly also generate virtual 3D models. While a considerably faster technique, it is incompatible with molecular labelling. In fact, use of accessible, cheap, highly specific, and versatile optical-based fluorescent markers is a key advantage in OPT and its benefits compensate for increased processing time. Two-photon microscopy, while also laborious, is likewise capable of 3D imaging of tissues using fluorescence. Remarkably, this technique can be used in living animals and therefore allow imaging of dynamic cellular interactions at high resolution^47^. Tissue penetration, however, can only be achieved up to 400 nm in depth, rendering it inaccessible for whole-depth volumetric imaging of most organs without endoscopic means. Furthermore, for any application outside of the skin, this technique requires competence in mouse surgery and ethical approval due to increased strain to the animals. Lastly, any fluorescent staining approach used for intravital two-photon imaging needs to be compatible with living animals. That includes usage of antibody-based stainings which may activate, deplete, or interfere with normal cell function; inaccessibility of intracellular targets by large molecules; and the impermeable nature of some endothelial linings to certain dyes—all limitations which OPT does not exhibit. Lastly, Positron Emission Tomography (PET) is another tool for 3D imaging of tissues, with the added advantages that it is non-invasive, and can be used longitudinally and for whole-body imaging in both live rodents and humans. While fludeoxyglucose (FDG)-PET is useful for imaging intestinal tumours in clinical practice, targeted PET imaging relies on the availability of radioisotope-labelled molecules which can selectively bind to target proteins, making the technique less accessible than OPT. Lastly, the resolution of PET is typically in the mm-scale, meaning that small structures such as ILFs would not be visible. All in all, OPT coupled with RevOPT can be a simple solution for simultaneous molecular and morphological volumetric imaging requiring precise pinpointing of ROIs.

Other potential drawbacks of OPT are possible artefacts due to tissue clearing. Solvent-based clearing methods are most appropriate for whole-mount immunostaining yet can also result in changes to the tissue. The process, based on delipidation and dehydration, results in complete clearing but can cause excessive autofluorescence due to haemoglobin, tissue shrinkage, or even tissue damage^48^. By implementing perfusion with PBS followed by PFA, one can remove residual blood sufficiently to diminish autofluorescence. On the other hand, others have successfully used OPT for notoriously autofluorescent tissues such as the mouse heart, with minimal interference and optimal leveraging of residual autofluorescence for imaging infarct injury^49^. Some shrinkage during dehydration and clearing is probably unavoidable. While shrinking due to different clearing protocols has been characterised by MRI for the brain^50^, to date little systematic work has been done for the intestine. Additionally, while RevOPT results in tissue rehydration, removed lipids cannot be replaced. The technique, as shown in the present manuscript, does however allow for downstream high-resolution microscopy with no apparent changes to morphology or tissue damage and maintaining original staining weeks after OPT.

Tissue autofluorescence serves multiple roles in the interpretation of microscopy images. It can provide crucial contextualization for fluorescent labels within tissues and facilitate the interpretation of functionality based on fluorescent signals. In addition, the intestinal architecture observed by autofluorescence imaging provides a label-free method for the characterization of diverse parameters^51^ which may be used as comprehensive measures of gut integrity and leakiness^52^. Such a technique could be applied to histopathologic scoring where structural deformation is symptomatic of disease. For example, coeliac disease (CD) is characterized by a destruction of the intestinal epithelium driven by gluten-activated inflammation, resulting in observable villous atrophy and lymphocytic infiltration of the epithelium, as shown recently in a novel mouse model of CD^53^. Current methods for the diagnosis of coeliac disease rely heavily on histological observations of prepared endoscopic biopsies, with a necessity for multiple collections due to non-homogeneous tissue alterations^54^. With the ability to accurately reconstruct mesoscale volumes, the presented optical projection tomography pipeline offers an alternative approach that maintains structural integrity whilst multiplying the field of view available for diagnostic observation.

The gastrointestinal tract constitutes an essential site for crosstalk between the external environment and the host, and which dictates immune development^55,56^. Gut immunity is implicated in intestinal diseases, such as inflammatory bowel disease^57^, and to systemic disorders ranging from metabolic diseases such as diabetes^54^ to neurodevelopmental, neuroinflammatory, and neurodegenerative diseases^56,58–60^. Thus, imaging and characterizing gut-associated lymphoid tissues (GALT) is important for understanding how immune development impacts health. Yet, the spatial distribution of immune structures in the gut is not well documented at the mesoscale. To our knowledge, our method is the first to image GALTs in 3D at a centimetre scale, with subsequent high resolution 2D ROI referencing.

In order to show the applicability of OPT to characterize the mesoscale organization of cell types within tissues, we explored the development of GALTs in models where age and the microbiota are manipulated. We stained the CD45 antigen that is found on hematopoietic cells^61^ from which almost all immune cell types are derived^62^. CD45-rich regions identified as isolated lymphoid follicles (ILF) were found in the gut of 30-week-old SPF C57BL/6J mice. ILFs are a sub-category of gut-associated lymphoid tissues^38^ whose functions are to limit contact between luminal microbiota and the epithelium via IgA secretion, and to sense epithelial breaching by bacteria and signal the need for phagocytosis to surrounding macrophages^63^. These are thought to depend on fibroblastic reticular cells (FRCs) and follicular dendritic cell (FDC)-like fibroblasts, to be seeded by Lymphoid Tissue inducer (LTi) cells, and to require microbiota-induced IL-25 and IL-23^64^. Yet the exact mechanism remains to be fully elucidated as well as their development characterized along long stretches of the intestine in 3D. For the first time, we are able to measure the variation in volume and the spatial distribution of ILFs in 3D throughout an uninterrupted section of tissue at the millimetre scale. This technique may prove useful in the tissue-wide imaging and characterisation of LTi cell clusters and their development into mature GALTs. Indeed, we demonstrate simultaneous acquisition in two separate channels with no abnormal overlap of fluorescence, meaning that multi-parameter fluorescent acquisitions can be achieved subject to correct combinations of detectors and light sources.

The age and microbiome dependence of the maturation and regulation of gut immune responses has become evident in recent years^65^. Initial exposure to a microbial environment during the neonatal period shapes the immune system throughout development^66^. In accordance with this, we find a sparse immune signal in adolescent SPF mice, with no discernible GALTs. In addition, the study of germ-free and gnotobiotic models has proven that the gut microbiota is necessary for the development of a mature and complete immune system^29^, with post-gnotobiotic colonization with commensal bacteria resulting in the acute induction of lymphoid tissue genesis^19^. Thus, the lack of isolated lymphoid follicles in the small intestine of adult germ-free mice observed here is indicative of the expected stunted immune system development in germ-free conditions. One limitation of the present study, however, is that only female mice were used across all groups. It is well accepted that sex differences exist, and that female immune responses in adults are often stronger than those of males. While an effect of sex on microbial composition was not readily discernible in adolescent mice^67^, it would be interesting to investigate differences in lymphoid structures throughout sexual development and in adults.

We also demonstrate the traceability of ROIs between imaging modalities by selecting ILFs in OPT reconstructions and performing RevOPT and confocal microscopy. The distances measured by image processing and by tracking the number of sectioning depths leads to an accurate correlation of signal localization. With signal density quantification, we are able to determine a limit of detection for regions of interest in OPT scans that may become a benchmark for the selection of targets of interest for OPT imaging. RevOPT adds value to our pipeline as it addresses the need for microscopic analysis of biological landscapes whilst offering the opportunity to interpret signals at the mesoscale.

OPT has been a powerful tool for 3D imaging for two decades, and its range of applications and performance continue to improve. We describe a methodology and application case for studying the immune compartment of the intestine, by combining multispectral imaging involving targeted immunostaining, chemical dyes, and natural properties of tissues. Logical next steps include the staining of multiple targets for deep immunophenotyping. While theoretically feasible and only limited by spectral overlap of emitters and detectors, multiple immunostaining has only been done for the brain^68^. For the intestine, this could prove useful for the characterisation of lymphoid structures during development and disease, and to elucidate the immune interface with the microbiome. Another aspect that may benefit widespread use of OPT imaging is automation and high-throughput screening. Yet, high-throughput OPT imaging of the intestine, in the conventional sense, is for now unfeasible due to the long tissue processing times. While multiple tissues may be processed simultaneously, incubation timings are required for efficent clearing. The high-throughput aspect of OPT relies in the extraction of high-volume information from ROIs across long segments of tissue, which is prohibitively impractical using conventional 2D imaging. Lastly, the prospect of extracting information and imaging a whole intestinal length per animal makes OPT an enticing system to use, with obvious advantages over other reductionist techniques. Indeed, we have previously imaged and stitched together sequential segments of up to 3 cm of intestine^27^, and theoretically this could be expanded to the entire gastrointestinal tract. For the adult intestine, this represents ca. 50 cm of tissue, achievable in about 10 stitching processes, including a 0.5 mm overlap between segments. This could unlock high-resolution volumetric information of about 1.5 m^2^ of intestinal tissue.

Collectively, we provide an imaging pipeline for versatile multi-modal imaging of the mouse intestine and its associated immune compartment. Furthermore, we demonstrate its ability to quantitatively characterise sparsely distributed structures throughout centimetre-long segments of the intestine, in volumetric terms. Cut-free sections reduce the presence of common artefacts that distort the sample and impede large-scale histopathological interpretations. The virtual sections also simplify the registration of multiple imaging depths for 3D or concatenated 2D segmentation of regions of interest, as we have shown here. Finally, we demonstrate the advantage of combining OPT with RevOPT for the empirical selection of regions of interest for high-resolution downstream imaging. We believe the ease of implementation and the resulting possibilities of analysis in large volumes and at high resolution make the gutOPT pipeline an attractive method for preclinical characterization of gut tissues in mice. Its potential for implementation in whole human tissue biopsy imaging raises exciting prospects for clinical diagnostics. Thus, gutOPT addresses the need for a detailed yet holistic approach to understanding the complex physiological interactions involved in gut health and disease.

## Methods

### Animal experimentation

Specific pathogen free female C57BL/6 J mice were purchased and housed at the École Polytechnique Fédérale de Lausanne (Switzerland) under specific pathogen free conditions with *ad libitum* access to food and water, according to guidelines and regulations of the state of Vaud, Switzerland (authorization VD3448). Adult and adolescent female mice used were 30- and 2-week old respectively. Female, age-matched, Germ-free C57BL/6 J mice were obtained from the Clean Mouse Facility, University of Bern (Switzerland). Germ-free status was routinely monitored by culture-dependent and -independent methods and confirmed to be microbial-free. Experiments were performed in accordance with regulations approved by the ethical and veterinary committee of the Canton of Vaud, Switzerland. As was described previously^27^, mice were deeply anesthetised by intra-peritoneal injection of 50 mg/kg sodium pentobarbital prior to a transcardiac perfusion of 10 ml heparinised PBS (5 I.U./ml Liquemin). Terminal ileum samples were fixed by 4% paraformaldehyde (CAS 30525-89- 4, Carl Roth AG 0964.1) overnight post-fixation step at 4 °C.

### Intestinal sample preparation

All the following steps took place in the dark. Samples were washed for 30 minutes in PBS after the overnight fixation. A 45-minute step-wise dehydration in methanol precedes overnight autofluorescence quenching in a 2:1:3 ratio solution of MetOH:DMSO:H_2_O_2_ overnight at room temperature. The samples are washed twice in pure MetOH in preparation for three freeze-thaw cycles between −80 °C and room temperature (1 hour and 30 minute cycles respectively) in order to permeabilize the tissue before antibody-mediated staining. A step-wise rehydration to TBS-Tween prepares the samples for antibody-mediated staining of targets. This begins with blocking for 24 h, is followed by a primary antibody incubation for 48 h and a 24 h washing step and ends with a 48 h incubation in a secondary antibody and a final 24 h washing step. To stain immune cells, we used 20 μg/mL of a rat anti-mouse CD45 monoclonal antibody conjugated to APC (BioLegend 147708) and 2 μg/mL of a goat anti-rat IgG (H + L) Alexa Fluor 647 (Invitrogen A21247). Samples were mounted in custom cylindrical molds in 1.5% agarose, dehydrated in pure methanol (CAS 67-56-1, Sigma-Aldrich 322415) for 24 hours and rehydrated in a 1:2 benzyl alcohol:benzyl benzoate mixture for a minimum of 48 h before acquisition (BA: CAS 100-51-6, Sigma- Aldrich 305197; BB: CAS 120-51-4, Sigma-Aldrich B6630).

### OPT imaging

Detailed descriptions of the optical setup are available at Schmidt, C. et al. (2021)^27^. Briefly, multi-channel sets of projections were acquired first with a 625 nm LED (filtered within a 595 nm and 645 nm range, AT620/50x, Chroma) for the far-red CD45-positive signal first, followed by a 415 nm LED (400–440 nm filter range, AT420/40x, Chroma) for tissue autofluorescence. In each channel, either 400 or 1200 projections were acquired, totalling between 4 to 10 minutes. Subsequently, reconstruction was performed using our previously described filtered back-projection algorithm and code. Volumetric reconstruction of each channel, and superimposition were assembled. At least 10 different sites were quantified per group. Reconstructed stacks were cropped using ImageJ.

### Reverse OPT

After OPT imaging, the samples were dehydrated in pure methanol and rehydrated in PBS for 24 h each. At this stage, it is possible to carefully track regions of interest identified in the 3D OPT images to be specifically observed using other imaging modalities downstream. The samples were extracted from the agarose molds and frozen in optimal cutting temperature (OCT) medium on dry ice. Using a Leica CM3050S cryostat, 25 micrometre-thick sections were collected and mounted on coated glass slides (Epredia™ J1800AMNZ). The sections were counterstained with DAPI (Thermofisher D1306) at a concentration of 5 μg/ml for 10 minutes with a 5 minute pre- and post-wash with 0.3% Triton-X100 in PBS.

### Confocal microscopy

Confocal microscopy was performed using a Leica SP8 inverted microscope, producing two-channel images encompassing the CD45-positive and nuclei signals. Sequential acquisition began with the AlexaFluor647 channel followed by the DAPI channel. Exposure times were determined according to live observation of pixel intensities in order to avoid over-exposure of the tissue. Tiled acquisitions of whole-gut sections were performed using the automated tile function in the LAS-X software.

### Virtual unfolding

The image processing pipeline for virtual unfolding was inspired by previous reports^28^ applied using Fiji^69^ and is available in the form of a macro algorithm (see Supplementary Code 1). All steps outlined below are applied to all of the sections within the filtered-back-projection stacks. First, the gut tissue is segmented from the lumen and surrounding background and a mask is created. For each section, the segmented tissue outline is added to the ROI manager. The centroid coordinates within the tissue outline are calculated and used as an origin for the identification of a sectioning origin at a 45° angle from the centre. From this point, we interpolate a polygon shape to draw the line along which the unfolding takes place. A stack of the straightened images is produced and re-sliced orthogonally to create the unfolded image whose field of view include the entire surface of the sample, spanning the lumen in the innermost slice to the outermost layers of the gut. From this image, the apex of each villus is identified by applying a Laplacian filter and extracting local maxima ROIs, whose density can then be calculated within a defined area.

### ILF segmentation

Quantifiable characteristics were extracted from Filtered Back Projection reconstructions showing the isolated lymphoid follicles using the surface tool in Imaris. The smoothing of surface areas was set to 2 μm and thresholding based on absolute intensity, whose values were set visually by the user. Larger structures were segmented by implementing the “number of voxels” filter. Volume and distance statistics were exported in csv format for data plotting.

### Statistics and Reproducibility

Data was analysed and comparisons and statistical testing was performed using Graphpad Prism 9. Datapoints are shown individually and data distribution shown using violin plots. Two-group comparisons were analysed using a two-tailed Student’s *t* test. Differences were considered significant at a *p* < 0.05 threshold. Specific *p*-values are shown, unless below *p* < 0.0001. The sample sizes and statistical comparison groups are indicated in the Figure legends.

### Reporting summary

Further information on research design is available in the Nature Portfolio Reporting Summary linked to this article.

## Supplementary information

Supplementary Information

Description of Additional Supplementary Data

Supplementary Data 1

Supplementary Movie 1

Reporting Summary

Supplementary Code 1

## Data Availability

The source data underlying the graphs and charts in Figs. 2–4 are provided in the Supplementary Data 1.
