# Supplementary material for: Optical imaging of the small intestine immune compartment across scales
Source: Commun Biol. 2023 Mar 31;6:352. doi: 10.1038/s42003-023-04642-3 (PMC10066397; doi:10.1038/s42003-023-04642-3)
Supplement: Supplementary file 1 — Supplementary Information [file 42003_2023_4642_MOESM1_ESM.pdf]

Supplementary Information

Optical Imaging of the Small Intestine Immune Compartment Across Scales

Arielle Louise Planchette[1]#, Cédric Schmidt[2], Olivier Burri[3], Mercedes Gomez de Agüero[4,5], Aleksandra Radenovic[1]#, Alessio Mylonas[1]*, Jérôme Extermann[2]*

#e-mail: arielle.planchette@epfl.ch and aleksandra.radenovic@epfl.ch

*These authors contributed equally

Affiliations

[1]Institute of Bioengineering, École Polytechnique Fédérale de Lausanne (EPFL), 1015, Lausanne, Switzerland
[2]HEPIA/HES-SO, University of Applied Sciences of Western Switzerland, Rue de la Prairie 4, 1202 Geneva, Switzerland
[3]BioImaging & Optics Platform, Ecole Polytechnique Fédérale de Lausanne (EPFL), 1015, Lausanne, Switzerland
[4]Host-microbial interactions group, Institute for Systems Immunology, Max Planck research group, University of Würzburg, Germany
[5]Mucosal Immunology Group, Department for Biomedical Research, University of Bern, Switzerland

Supplementary information content

Supplementary Figure 1 : Segmentation and Unfolding of OPT filtered back projections of mouse intestine

Title: Supplementary Movie 1. Representative movie of a rendered three-dimensional image acquired and processed by optical projection tomography, of samples shown in Fig. 3 (a) and (d). 3D image consists of two channels, the autofluorescent signal (cyan) and isolated lymphoid follicles stained with an anti-Cd45 antibody (magenta).

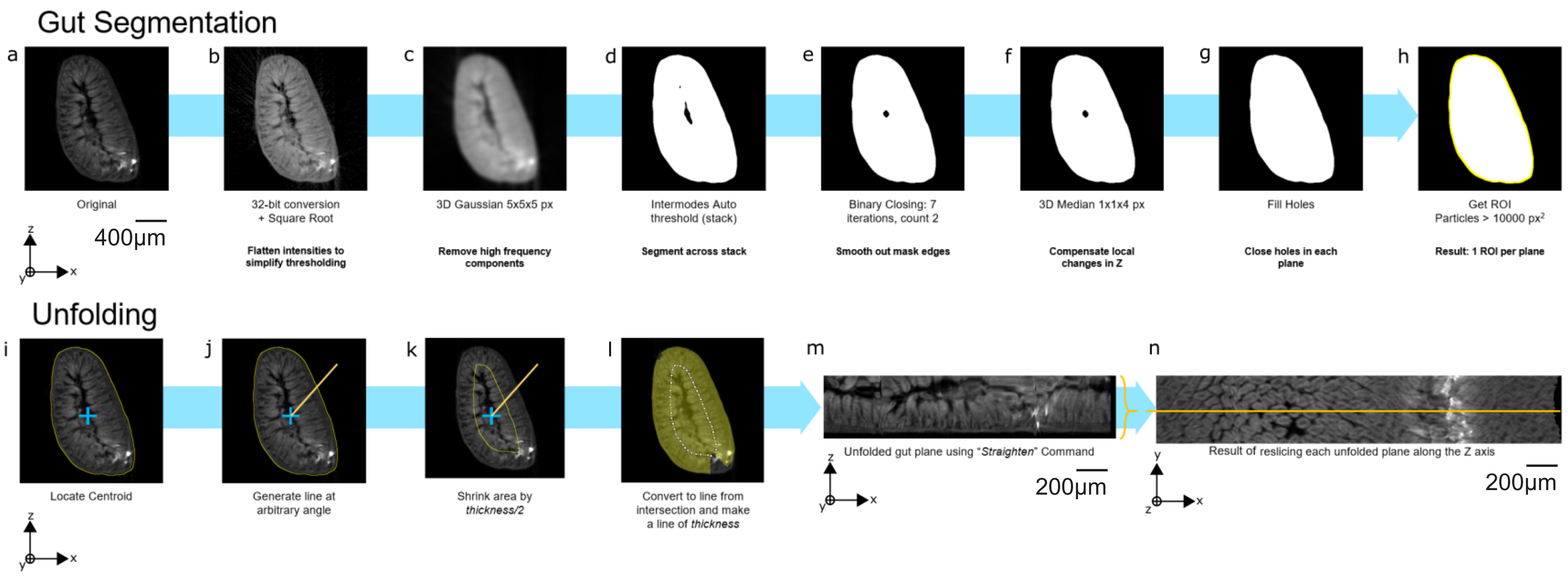

Supplementary Figure 1 : Segmentation and Unfolding of OPT filtered back projections of mouse intestine

Image processing is performed in ImageJ, using a macro written by the authors. (a-h) Initial segmentation of the gut sample and background throughout the depths of the imaged sample. (i-n) Unfolding and reslicing of the intestinal lining, starting from the centroid. (m) top view of unfolded sample. (n) orthogonal view from within the lumen, making villi cross sections visible.
