# Supplementary material for: Optical imaging of the small intestine immune compartment across scales
Source: Commun Biol. 2023 Mar 31;6:352. doi: 10.1038/s42003-023-04642-3 (PMC10066397; doi:10.1038/s42003-023-04642-3)
Supplement: Supplementary file 2 — Description of Additional Supplementary Data [file 42003_2023_4642_MOESM2_ESM.docx]

**Description of Additional Supplementary Files**

**File name:** Supplementary Movie 1

**Description:** Representative movie of a rendered three-dimensional image acquired and processed by optical projection tomography, of samples shown in Fig. 3 (a) and (d). 3D image consists of two channels, the autofluorescent signal (cyan) and isolated lymphoid follicles stained with an anti- CD45 antibody (magenta).

**File name:** Supplementary Data 1

**Description:** Numerical source data.

**File name:** Supplementary Code 1

**Description:** Fiji macros used to perform virtual unfolding.
