# Supplementary material for: Optical imaging of the small intestine immune compartment across scales
Source: Commun Biol. 2023 Mar 31;6:352. doi: 10.1038/s42003-023-04642-3 (PMC10066397; doi:10.1038/s42003-023-04642-3)
Supplement: Supplementary file 5 — Reporting Summary [file 42003_2023_4642_MOESM5_ESM.pdf]

nature portfolio

Corresponding author(s): Arielle Planchette and Aleksandra Radenovic

Last updated by author(s): 11/11/2022

Reporting Summary

Nature Portfolio wishes to improve the reproducibility of the work that we publish. This form provides structure for consistency and transparency in reporting. For further information on Nature Portfolio policies, see our Editorial Policies and the Editorial Policy Checklist.

Statistics

For all statistical analyses, confirm that the following items are present in the figure legend, table legend, main text, or Methods section.

n/a	Confirmed	
☐	☒	The exact sample size (*n*) for each experimental group/condition, given as a discrete number and unit of measurement
☒	☐	A statement on whether measurements were taken from distinct samples or whether the same sample was measured repeatedly
☐	☒	The statistical test(s) used AND whether they are one- or two-sided<br>*Only common tests should be described solely by name; describe more complex techniques in the Methods section.*
☒	☐	A description of all covariates tested
☒	☐	A description of any assumptions or corrections, such as tests of normality and adjustment for multiple comparisons
☐	☒	A full description of the statistical parameters including central tendency (e.g. means) or other basic estimates (e.g. regression coefficient) AND variation (e.g. standard deviation) or associated estimates of uncertainty (e.g. confidence intervals)
☒	☐	For null hypothesis testing, the test statistic (e.g. *F*, *t*, *r*) with confidence intervals, effect sizes, degrees of freedom and *P* value noted<br>*Give P values as exact values whenever suitable.*
☒	☐	For Bayesian analysis, information on the choice of priors and Markov chain Monte Carlo settings
☒	☐	For hierarchical and complex designs, identification of the appropriate level for tests and full reporting of outcomes
☒	☐	Estimates of effect sizes (e.g. Cohen's *d*, Pearson's *r*), indicating how they were calculated

Our web collection on statistics for biologists contains articles on many of the points above.

Software and code

Policy information about availability of computer code

Data collection	Data collection protocol is explained in manuscript entitledHigh resolution optical projection tomography platform for multispectral imaging of the mouse gut DOI: https://doi.org/10.1364/BOE.423284
Data analysis	Data analysis pipeline is explained in manuscript entitledHigh resolution optical projection tomography platform for multispectral imaging of the mouse gut DOI: https://doi.org/10.1364/BOE.423284

For manuscripts utilizing custom algorithms or software that are central to the research but not yet described in published literature, software must be made available to editors and reviewers. We strongly encourage code deposition in a community repository (e.g. GitHub). See the Nature Portfolio guidelines for submitting code & software for further information.

Data

Policy information about availability of data

All manuscripts must include a data availability statement. This statement should provide the following information, where applicable:

- Accession codes, unique identifiers, or web links for publicly available datasets
- A description of any restrictions on data availability
- For clinical datasets or third party data, please ensure that the statement adheres to our policy

Data and code underlying the results presented in this paper are available on website srm.epfl.ch.

Human research participants

Policy information about studies involving human research participants and Sex and Gender in Research.

Reporting on sex and gender	NA
Population characteristics	NA
Recruitment	NA
Ethics oversight	NA

Note that full information on the approval of the study protocol must also be provided in the manuscript.

Field-specific reporting

Please select the one below that is the best fit for your research. If you are not sure, read the appropriate sections before making your selection.

☒ Life sciences ☐ Behavioural & social sciences ☐ Ecological, evolutionary & environmental sciences

For a reference copy of the document with all sections, see nature.com/documents/nr-reporting-summary-flat.pdf

Life sciences study design

All studies must disclose on these points even when the disclosure is negative.

Sample size	NA
Data exclusions	No data exclusion
Replication	NA this is imaging project and not applicable in our view although we imaged multiple samples
Randomization	NA
Blinding	NA

Reporting for specific materials, systems and methods

We require information from authors about some types of materials, experimental systems and methods used in many studies. Here, indicate whether each material, system or method listed is relevant to your study. If you are not sure if a list item applies to your research, read the appropriate section before selecting a response.

Materials & experimental systems

n/a	Involved in the study	
☐	☒	Antibodies
☒	☐	Eukaryotic cell lines
☒	☐	Palaeontology and archaeology
☐	☒	Animals and other organisms
☒	☐	Clinical data
☒	☐	Dual use research of concern

Methods

n/a	Involved in the study	
☒	☐	ChIP-seq
☒	☐	Flow cytometry
☒	☐	MRI-based neuroimaging

Antibodies

Antibodies used	rat anti-mouse CD45 (BioLegend 147708)<br>goat anti-rat IgG (H+L) Alexa Fluor 647 (Invitrogen A21247)
Validation	From BioLegend's website: "Each lot of this antibody is quality control tested by immunofluorescent staining with flow cytometric analysis. For flow cytometric staining, the suggested use of this reagent is ≤0.25 µg per million cells in 100 µl volume. It is recommended that the reagent be titrated for optimal performance for each application. Additional reported applications (for the relevant formats) include: immunohistochemical staining of paraffin embedded sections1 and frozen tissue sections2."

1. Kliment C, et al. 2009. J. Mol. Cell Cardiol. 47:730. (IHC)
2. Reynolds JM, et al. 2007. J. Immunol. 179:313. (IHC)

Animals and other research organisms

Policy information about studies involving animals; ARRIVE guidelines recommended for reporting animal research, and Sex and Gender in Research

Laboratory animals	Mus musculus, C57BL/6J, 14 days old or 30+ weeks old.
Wild animals	The study did not involve wild animals.
Reporting on sex	Findings apply only to female mice. It is known that immune responses are often stronger in females than males, yet sex does not influence the overall presence or absence of ILFs in adult mice. We conducted the study selectively in sex-matched conditions, because it is beyond the scope of the current study to pinpoint potential differences in ILF composition in males and females.
Field-collected samples	The study did not involve samples collected from the field.
Ethics oversight	Ethical approval was obtained from the cantonal veterinary office of Vaud, Switzerland under authorization number VD3448.

Note that full information on the approval of the study protocol must also be provided in the manuscript.
